# ECMO use and mortality in adult patients with cardiogenic shock: a retrospective observational study in U.S. hospitals

**DOI:** 10.1186/s12873-018-0171-8

**Published:** 2018-07-04

**Authors:** Rayan El Sibai, Rana Bachir, Mazen El Sayed

**Affiliations:** 1 Department of Emergency Medicine, American University of Beirut Medical Center, P.O. Box - 11-0236, Riad El Solh, Beirut, 1107 2020 Lebanon; 2 Emergency Medical Services and Prehospital Care Program, American University of Beirut Medical Center, Beirut, Lebanon

**Keywords:** Extracorporeal circulation, Extracorporeal membrane oxygenation, Shock, cardiogenic, Emergency service, hospital, Resuscitation, Critical illness

## Abstract

**Background:**

Extracorporeal membrane oxygenation (ECMO) is increasingly used in resuscitation of critically ill patients with documented improved survival. Few studies describe ECMO use in cardiogenic shock. This study examines ECMO use and identifies variables associated with mortality in patients treated for cardiogenic shock in US hospitals.

**Methods:**

A retrospective observational study of the US Nationwide Emergency Department Sample (NEDS) database of 2013 was conducted. Weighted visits for cardiogenic shock (discharge diagnosis) with ECMO use were included. Collected data was analyzed and variables associated with mortality were identified.

**Results:**

A total of 922 weighted patients with cardiogenic shock and ECMO were included. Mean age was 50.8 years. They were more commonly males (66.3%; *n* = 658). Slightly over half (51.0%, *n* = 506) survived to hospital discharge. Mean charges per patient were $589,610.5. Mean length of stay was 21.8 days.

Increased mortality was associated with presence of respiratory diseases (OR = 3.83), genitourinary diseases (OR = 4.97), undergoing an echocardiogram (OR = 4.63), and presenting during seasons other than Fall. Lower mortality was noted in patients with injury and poisoning (OR = 0.47), in those who underwent certain vascular procedures (OR = 0.49) and those with increasing length of stay (OR = 0.90).

**Conclusion:**

Mortality in patients with cardiogenic shock remains high despite ECMO use. Season of admission (other than Fall) and presence of specific comorbidities (Respiratory and genitourinary diseases) are associated with increased mortality in this population. Familiarity with these variables can help identify patients at higher risk of death and can help improve outcomes further in cardiogenic shock.

**Electronic supplementary material:**

The online version of this article (10.1186/s12873-018-0171-8) contains supplementary material, which is available to authorized users.

## Background

Mechanical cardiopulmonary assistance, which is often utilized as an integral part of certain invasive cardiac surgeries, is increasingly used in resuscitation of patients in intensive care settings and emergency departments [1–12]. Prolonged cardiopulmonary assistance is called extracorporeal membrane oxygenation (ECMO) or extracorporeal life support (ECLS) and is a sophisticated and resource-intensive intervention for critically ill patients with cardiac or respiratory dysfunction [11, 12]. ECMO and ECLS are often used interchangeably [13].

There are two types of ECMO – veno-venous (VV) and veno-arterial (VA) ECMO – depending on differences in vascular access. During VV ECMO, blood is extracted from the vena cava or right atrium and returned oxygenated to the right atrium. During VA ECMO, blood is extracted from the right atrium and returned oxygenated to the arterial system, most commonly the iliac artery. VA ECMO provides hemodynamic support in addition to respiratory support [11].

Although ECMO use is more commonly described in the pediatric population, ECMO use in adults for cardiac and respiratory indications more recently became the largest contributing group in the Extracorporeal Life Support Organization Registry, which was established to improve quality and outcome of ECMO use [4, 12]. ECMO is used for various cardiac or pulmonary diseases including: post-cardiotomy, myocarditis, acute coronary syndrome or refractory cardiac arrest, as a bridge to heart/lung transplant, cardiogenic shock, respiratory failure, trauma/drowning as well as other conditions [14]. In 2015, cardiogenic shock was found to be the most common diagnosis associated with ECLS use in adults with VA ECMO, occurring in 60.7% of such patients [4]. Cardiogenic shock is a clinical condition of inadequate tissue perfusion due to cardiac dysfunction that is associated with persistent hypotension and end organ damage. Cardiogenic shock often results from myocardial infarction but can also result from other cardiac diseases such as myocarditis, pericarditis, and cardiomyopathy [15].

ECMO use improves survival in patients with cardiac disease with survival rates ranging from 20 to 50% [1–10]. ECMO also increases 30-day survival rates when used to bridge patients in cardiogenic shock to coronary intervention procedures [16]. Additionally, ECMO improves outcomes in patients with cardiac arrests – mainly survival without neurologic deficit – when added to cardiopulmonary resuscitation (CPR) [3, 17].

Contraindications for VA ECMO in cardiac failure do exist and include the following: ethical considerations and patient’s will, no bridging goal, severe aortic regurgitation, aortic dissection, severe peripheral artery disease (iliac), left ventricular thrombus (relative contraindication) [18]. Despite several observational and registry studies on ECMO use, few studies describe the extent of ECMO use in patients with cardiogenic shock. This study examines cardiogenic shock presentations that involved ECMO use and identifies variables associated with mortality in this U.S. hospital population sample.

## Methods

### Data and materials

NEDS is a product of Healthcare Cost and Utilization Project (HCUP) under the auspices of the Agency for Healthcare Research and Quality (AHRQ) [19]. It is the largest U.S. all-payer ED database, combining medical and non-clinical data from both national and state sources. Data from this stratified sample is accrued from 945 hospital-owned EDs located across 30 States and the District of Columbia, reflecting approximately 20% of all hospital-based EDs located in the United States. The following stratification variables were used to weight data per HCUP specifications: U.S. Census region, urban-rural location, ownership, and teaching status of the hospital as well as trauma center designation [20]. This stratified sample of patients is statistically weighted using the above stratification variables to approximate and examine national estimates. Weighting of sample compensates for different probabilities of selection as part of the dataset sample. As per requirements for publishing HCUP data and to safeguard patients’ privacy, data on any variable with size less than or equal to 10 are excluded.

### Study design and population

This retrospective study used the 2013 public release dataset of the US Nationwide Emergency Department Sample (NEDS) database. The 2013 NEDS dataset contains information on weighted 134,869,015 ED visits. Patients were included in this study if they met the following criteria: Adult patients (age 18 or older) who presented with cardiogenic shock and underwent ECMO procedure and died in the ED or were admitted to the same hospital during the selected visit.

### Data definitions

The NEDS data elements include: demographic patient information; mechanism, intent, and severity of injury; admission and discharge status; payment source; healthcare expenses and general hospital characteristics. The AHRQ Clinical Classifications Software (CCS) was used to select all patients with cardiogenic shock. CCS is a tool for grouping patient diagnoses and procedures into a convenient number of clinically meaningful categories similar and equivalent to than the International Classification of Diseases (ICD) coding system.

The following CSS codes are specific for cardiogenic shock: CCS 97, CCS 100, CCS 101, CCS 103, CCS 106, CCS 107, CCS 108 [12]. A list of Equivalent ICD-9 CM codes and variable classification is included (See attachment, Additional File 1 which illustrates code and variable classifications). Additionally, the ICD-9 CM procedure code 39.65 was used to gather all patients who underwent ECMO procedure.

Chronic diseases and procedures with frequencies less than 10% were removed from the analysis and were not displayed results. Injury severity score (ISS) is considered as the gold standard trauma severity score to predict morbidity, increased hospital stay and mortality in trauma patients. An ISS score greater than 15 is historically used as a cutoff value for severe trauma injury [21]. This study used a more conservative cut off value for ISS of > 8 since very few patients had an ISS score greater than 15. Previous studies have also shown that increasing injury severity (ISS of > 8) is associated with increased hospital stay and ICU admission [22].

AHRQ provides a database and a software tool, called the Chronic Condition Indicator (CCI), that allows for the identification of chronic diseases using ICD-9 CM coding. CCI was utilized in this study for the purposes of investigating presence of chronic conditions according to body system categories affected. According to CCI, the definition of a chronic disease is “a medical condition which lasts for twelve months or longer with either one of the following criteria being met: (1) the medical condition restricts self-care, independent living, and social interaction; (2) the medical condition results in the need of ongoing medical intervention.”

Additionally, NEDS categorizes ICD-9 CM procedure codes into four broad categories labeled as either major or minor and therapeutic or diagnostic. An ICD-9 CM procedure is classified as “minor if the procedure is a non-operating room procedure and major if the procedure is a valid operating room procedure by the Diagnosis Related Group (DRG) grouper” (an ICD-9 CM grouping system). CCS procedure codes were also used to examine most common procedures other than ECMO.

### Statistical analysis

IBM-SPSS 24 was used to carry out descriptive analysis on the socio-demographic, clinical and hospital characteristics. Mean with associated 95% confidence intervals (CI) was used for continuous variables, while frequencies, percentages and 95% CI were used for categorical variables.

The standard statistical methods that treat the data as being emerged from a simple random sampling data were inappropriate for the NEDS survey design and would yield biased estimates, overly-narrow confidence levels and misleading significance tests due to the type I error generated from the biased results. The Rao-Scott chi-square test, a modified version of the Pearson’s chi-square test, as well as a general linear model (GLM) for complex sampling (CS) were used to assess the significance of the statistical association of the independent categorical and continuous variables respectively between the two groups (those who survived and those who did not). Logistic regression was conducted to identify significant associations with mortality in ECMO patients with cardiogenic shock. Variables that were significantly different between the two groups when compared by outcome (mortality) were included in the multivariate analysis.

All estimates (means, percentages, odds ratio and confidence intervals) were drawn by complex sampling methods, particularly, CSTABULATE, CSDESCRIPTIVES, CSGLM and CSLOGISTIC to adjust for the NEDS survey design and to ensure the results are accurate. CSTABULATE is a complex sample procedure that produces analysis for categorical variables. CSDESCRIPTIVES and CSGLM are two different complex sample procedures that allow for analysis of continuous variables at the univariate and bivariate levels respectively. Lastly, the Complex Samples Logistic Regression procedure, CSLOGISTIC, performs logistic regression for samples drawn by complex sampling methods.

Complex sampling design of the study resulted in decimal places among sample frequencies. To avoid rounding errors and for the sake of clarity, variables were restored to discrete format by discarding decimals. In addition, a maximal value for total sample frequency was capped at 992 patients. Analysis was verified using *HCUPnet*, a free on-line query system based on data from HCUP [23]. Lastly, a *p*-value of < 0.05 was used to indicate statistical significance.

## Results

A total of 8,605,807 weighted adult visits were admitted to the emergency department with cardiogenic shock. Of those, 992 weighted adult patient visits included an ECMO procedure yielding a rate of 0.1 per 1000 ED visits for cardiogenic shock.

The mean age of the study population was 50.8 years (95%CI 48.8–52.7). Patients were more commonly males (66.3%; 95%CI 60.3–71.8). Visits were equally distributed across all four seasons and were mainly during the weekday (Monday through Friday) (70.6%, *n* = 700). Over half of visits were to large metropolitan hospitals (fringe 39.4%, *n* = 389 and central 29.1%, *n* = 287). Private insurance (47.8%, *n* = 472) as well as Medicare and Medicaid (43.6%, *n* = 430) were the most common primary expected payer types. (Table 1).

**Table 1:** Characteristics of Study Population

Continuous Variables	Range	Median (IQR)	Mean (95% CI)
Age (years)	18–90	54 (23)	50.8 (48.8–52.7)
*Categorical Variables*		*Count (N = 992)*	*Percent (95% CI)*
Gender (male)		658	66.3 (60.3–71.8)
Median household income^*a*^			
$1 - $37,999		126	13.2 (9.8–17.6)
$38,000 - $47,999		238	25.1 (19.9–31.0)
$48,000 - $63,999		238	25.0 (20.2–30.7)
$64,000 or more		348	36.7 (31.1–42.6)
Season of admission			
Winter		223	23.2 (18.3–29.0)
Spring		244	25.4 (20.5–31.0)
Summer		259	27.0 (21.8–33.0)
Autumn		234	24.3 (19.5–29.9)
Admission day			
Monday - Friday		700	70.6 (64.7–75.8)
Saturday - Sunday		292	29.4 (24.2–35.3)
Patient location^b^			
Large central metropolitan		287	29.1 (24.4–34.3)
Large fringe metropolitan		389	39.4 (34.5–44.5)
Medium metropolitan		147	14.9 (11.5–19.0)
Small metropolitan		66	6.6 (4.1–10.7)
Micropolitan		51	5.1 (3.2–8.1)
Not metropolitan or micropolitan		48	4.9 (3.0–7.9)
Primary expected payer			
Private insurance		472	47.8 (41.6–54.1)
Medicare & Medicaid		430	43.6 (37.3–50.1)
Self-pay		60	6.1 (3.8–9.7)
Other		25	2.5 (1.1–5.5)

^a^According to national quartile for patient ZIP code derived from ZIP Code-demographic data obtained from Claritas

^b^According to National Center for Health Statistics (NCHS) Urban-rural code

All patients had diseases of the circulatory system, and most patients had diseases of the respiratory system (82.7%, *n* = 820) as well as endocrine, nutritional, and metabolic diseases/immunity disorders (80.1%, *n* = 795) (Table 2). Few patients had injury diagnosis reported on record (14.2%, *n* = 141). The most frequent injury reported was suffocation injury (1.9%, *n* = 19) followed by falling injury (1.5%, *n* = 15). Only 5.3% (*n* = 52) had increased injury severity defined by an ISS of 9 or greater. The mean for total charges (both ED and inpatient services) was $589,610.5 (95% CI 512,270.0 – 666,950.9). The mean hospital length of stay was 21.8 days (95% CI 19.0–24.6).

**Table 2:** Chronic Conditions, Injuries, and Outcomes of Sample Patients

Categorical Variables			Count (*N* = 992)	Percent (95% CI)
Chronic conditions body system indicator				
Circulatory system			992	100
Respiratory system			820	82.7 (77.5–86.9)
Endocrine/nutritional/metabolic/immunity			795	80.1 (74.7–84.6)
Symptoms, signs, and ill-defined conditions			779	78.5 (73.1–83.1)
Genitourinary system			724	72.9 (67.1–78.1)
Blood and blood-forming organs			636	64.1 (58.0–69.7)
Injury and poisoning			617	62.2 (56.3–67.7)
Digestive system			414	41.8 (35.8–47.9)
Infectious and parasitic disease			393	39.7 (33.7–45.9)
Health status/contact with health services factors			383	38.6 (33.1–44.4)
Nervous system and sense organs			313	31.5 (26.1–37.5)
Mental disorders			186	18.8 (14.3–24.2)
Injury diagnosis reported on record				
No injury diagnoses reported			851	85.8 (80.8–89.7)
Injury is reported			141	14.2 (10.3–19.2)
Injuries Specified by the NEDS Database				
Injury by suffocation			19	1.9 (0.8–4.4)
Injury by falling			15	1.5 (0.5–4.1)
More than one injury diagnosis				
One or no injury diagnosis reported			938	94.5 (90.9–96.7)
More than one injury diagnosis			54	5.5 (3.3–9.1)
Unintentional injury indicated				
Intention not specified			924	93.1 (89.2–95.6)
Unintentional injury			68	6.9 (4.4–10.8)
Injury severity score^a^				90.3 (85.9–93.5)
No injury severity score			896	4.4 (2.4–7.8)
1–8			44	5.3 (3.0–9.0)
9–75			52	
Disposition of patient				
Admitted to same hospital			992	100
Survival to discharge			506	51.0 (44.8–57.1)
Died in the ED/hospital			486	49.0 (42.9–55.2)
Continuous Variables	Frequency	Range	Median (IQR)	Mean (95% CI)
Total charges^b^ (USD$)	988	3773–4,292,990	409,138 (468,598)	589,610.5 (512,270.0 – 666,950.9)
Length of stay (days)	992	0–131	16 (24)	21.8 (19.0–24.7)

^a^Assigned by ICPIC Stata program

^b^Total charges are for accumulated ED and Inpatient services

In addition to ECMO, patients with cardiogenic shock underwent different types of procedures (Table 3). By procedure class, all patients had major therapeutic procedures on record since ECMO procedure is categorized as major therapeutic. Most patients (89.1%, *n* = 884) also had minor therapeutic procedures. The most common procedure other than ECMO was respiratory intubation and mechanical ventilation (56.7%, *n* = 562).

**Table 3:** Extracorporeal Membrane Oxygenation Among Cardiogenic Shock: Procedures

Procedure class for inpatient	Count (*N* = 992)	Percent (95% CI)
Minor diagnostic	534	53.8 (47.8–59.7)
Minor therapeutic	884	89.1 (84.6–92.4)
Major diagnostic	62	6.2 (3.8–10.1)
Major therapeutic	992	100
Specific procedures using CCS codes	Count (*N* = 992)	Percent (95% CI)
Circulator system procedures		
Extracorporeal circulation auxiliary to open heart procedures^a^	992	100
Other operating room heart procedures^b^	510	51.4 (45.3–57.5)
Diagnostic cardiac catheterization; coronary arteriography	231	23.3 (18.6–28.9)
Conversion of cardiac rhythm	183	18.4 (14.4–23.3)
Heart valve procedures	179	18.1 (13.9–23.1)
Coronary artery bypass graft (CABG)	150	15.1 (11.5–19.6)
Other non-operating room therapeutic cardiovascular procedures	123	12.4 (9.0–16.9)
Percutaneous transluminal coronary angioplasty (PTCA)	113	11.4 (8.1–15.8)
Echocardiogram	107	10.8 (7.6–15.3)
Swan-Ganz catheterization for monitoring	102	10.2 (7.2–14.4)
Blood transfusion	220	22.2 (17.9–27.2)
Respiratory system procedures		
Respiratory intubation and mechanical ventilation	562	56.7 (50.5–62.7)
Other operating room procedures on respiratory system and mediastinum	182	18.4 (14.0–23.7)
Diagnostic bronchoscopy and biopsy of bronchus	135	13.6 (10.0–18.3)
Tracheostomy; temporary and permanent	111	11.2 (7.8–15.9)
Other procedures		
Other operating room procedures^b^	365	36.8 (31.1–42.9)
Other vascular catheterization; not heart	330	33.3 (27.8–39.3)
Other therapeutic procedures^c^	207	20.9 (16.5–26.1)

^a^This CCS procedure code also includes ICD-9 procedure code 39.65 for ECMO procedures

^b^Examples include operations on valves and septa of the heart, heart vessels, and the heart and pericardium

^c^Specifically procedures on vessels other than head/neck

^d^Nervous, ophthalmic, gastrointestinal procedures not otherwise specified

For Patients who underwent ECMO, there were significant differences between those who survived to hospital discharge and those who did not. Table 4 lists only the statistically significant variables between the two groups and which included age, season of admission, presence of certain chronic conditions and certain procedures, whether injury was reported, total charges, and length of stay (LOS). Those who survived were slightly younger than those who did not (49.5 vs 52.1, years respectively *p* = 0.009) and had significantly higher total hospital charges for services as well as total length of stay.

**Table 4:** Comparison of Study Groups Stratified by Outcome (Mortality)

	Survival to Discharge (*N* = 506)		Mortality (*N* = 486)		
Categorical Variables^a^	Count	Percent (95% CI)	Count	Percent (95% CI)	*p*-value
Season of admission					
Winter	85	17.4 (11.7–25.2)	138	29.2 (21.8–37.8)	0.006
Spring	104	21.3 (15.0–29.1)	140	29.7 (22.2–38.5)	
Summer	140	28.7 (21.3–37.5)	119	25.2 (18.2–34.0)	
Autumn	159	32.6 (25.6–40.3)	75	15.9 (10.3–23.7)	
Chronic conditions	506	100	487	100	
Respiratory system	389	76.9 (69.1–83.2)	431	88.6 (81.4–93.3)	0.014
Injury and poisoning	375	74.2 (65.9–81.0)	242	49.7 (41.1–58.3)	< 0.001
Genitourinary system	305	60.4 (51.7–68.4)	419	86.0 (79.6–90.7)	< 0.001
Digestive system	178	35.3 (27.5–43.9)	236	48.5 (39.8–57.3)	0.034
Nervous system & sense organs	127	25.1 (18.4–33.4)	186	38.2 (30.1–46.9)	0.025
Injury diagnosis					
No injury diagnoses	392	77.7 (69.3–84.3)	459	94.3 (88.7–97.2)	< 0.001
Injury is reported	113	22.3 (15.7–30.7)	28	5.7 (2.8–11.3)	
*Procedures*					
Other operating room procedures^b^	225	44.5 (36.0–53.3)	140	28.8 (21.4–37.6)	0.012
Tracheostomy^c^	82	16.1 (10.5–23.9)	30	6.1 (3.0–12.1)	0.015
Echocardiogram	36	7.1 (3.8–12.9)	71	14.7 (9.5–22.0)	0.05
Continuous Variables^d^	Count	Mean (95% CI)	Count	Mean (95% CI)	*p*-value
Age (years)	506	49.5 (46.8–52.1)	487	52.1 (49.3–54.9)	0.009
Total charges^e^ (USD)	501	695,089.0 (560,971.1-829,206.8)	487	480,927.5 (398,015.8-563,839.2)	< 0.001
Length of stay (days)	506	28.8 (24.4–33.1)	487	14.6 (11.5–17.6)	< 0.001

^a^The *p*-values for the categorical variables were calculated using the Rao-Scott chi-square test of the complex sample procedure

^b^Specifically procedures on vessels other than head and neck

^c^Temporary and permanent

^d^The *p*-values for the continuous variables were calculated using the general linear model of the complex sample procedure

^e^Total charges are for accumulated ED and Inpatient services

Table 5 shows the variables associated with mortality in ECMO patients. Having diseases of either the respiratory system (OR = 3.83, 95%CI 1.76–8.31) or genitourinary system (OR = 4.97, 95%CI 2.39–10.33) as well as undergoing an echocardiogram (OR = 4.63, 95%CI 1.300–16.504) were significantly associated with increased mortality. Higher mortality was also associated with visits during all seasons when compared to autumn.

**Table 5:** Mortality with ECMO use in Cardiogenic Shock^a^

	Odds Ratio	95% CI	*p*-value
CCI^b^ - Nervous system and sense organs diseases	1.22	0.64–2.32	0.541
CCI - Respiratory system diseases	3.83	1.76–8.31	0.001
CCI - Digestive system diseases	1.72	0.82–3.61	0.150
CCI - Genitourinary system diseases	4.97	2.39–10.33	< 0.001
CCI - Injury and poisoning	0.47	0.24–0.94	0.032
Injury diagnosis reported	0.45	0.15–1.36	0.156
Tracheostomy; temporary and permanent	0.46	0.14–1.48	0.190
Other operating room procedures^c^	0.49	0.24–1.00	0.048
Echocardiogram	4.63	1.30–16.50	0.018
Season of admission (Autumn)			
Winter	8.85	3.39–23.11	< 0.001
Spring	5.44	2.16–13.73	< 0.001
Summer	4.02	1.54–10.46	0.005
Age (1 year)	1.01	1.00–1.04	0.239
Length of Stay (1 day)	0.94	0.90–0.98	0.003
Total charges^d^ (per $10,000)	1.01	1.00–1.02	0.069

^a^Variables included in the model are: significant chronic conditions and procedures found at the bivariate level, injury diagnosis, season of admission, age, and total charges

^b^CCI denotes body system categories affected by chronic conditions

^c^Specifically procedures on vessels other than head and neck

^d^Total charges are for accumulated ED and Inpatient services

Conversely, having a chronic disease of injury and poisoning (OR = 0.382, 95%CI 0.191–0.765), undergoing other operating room procedures on vessels other than head and neck (OR = 0.49, 95%CI 0.24–1.00), as well as increasing total charges (OR = 0.993 95%CI 0.988–0.999) were associated with decreased mortality.

## Discussion

ECMO use is increasing in adult patients with cardiogenic shock, but few studies that describe ECMO use or associations with mortality in this population are available. This study is the first to use the largest US ED database, NEDS, to describe socio-demographic, clinical, and hospital characteristics for patients with cardiogenic shock who underwent ECMO.

This study also described patterns of ECMO use and outcomes of patients with cardiogenic shock. ECMO was used in 0.1 per 1000 ED/hospital visits with cardiogenic shock in 2013. Survival to hospital discharge was 51%. This finding is consistent with survival rates from previous studies that range from 20 to 50% [1–10].

The mean age of patients who presented to US EDs with cardiogenic shock and who underwent ECMO was 50.8 years (95%CI 48.4–52.7). Previous studies have shown similar characteristics [12, 24]. Maxwell et al. examined national ECMO use between the years 1998 to 2009 and found an overall mean age of 53.9 ± 0.4 years and a mean age of 48.9 ± 0.8 years among those with cardiogenic shock specifically [12]. Schmidt et al., on the other hand, analyzed ECMO use only for refractory cardiogenic shock between the years of 2003 and 2013 and found a mean age of 54 years (range 39–64) [24].

ECMO use as expected was mostly in large metropolitan hospitals. ECMO is considered a complex procedure and is fraught with complications such as stroke, neurologic complications, lower extremity ischemia, fasciotomy or compartment syndrome, amputation, acute kidney injury, renal replacement therapy, significant infection, major or significant bleeding and re-thoracotomy for bleeding or tamponade [14, 18]. Thus, it is usually reserved for when conventional treatments have failed and is usually undertaken in larger specialized centers.

Seasonal variation in mortality among patients presenting with cardiogenic shock and undergoing ECMO procedure was also identified in this study. The association of seasonal patterns on both hospital admission and mortality has been previously examined and described among patients with cardiovascular disease. Previous studies have reported increased adverse effects (hospital admission and mortality) during colder months (up to 10–20% increased mortality) [25–27]. Similarly, our study shows that the winter season was associated with the highest mortality (OR: 8.85; 95%CI: 3.39–23.11). Reasons for these seasonal variations though not directly identified in our study are multiple and complex, involving both physiologic and behavioral causes. Previously identified causes include sudden cold acclimatization, change in dietary patters, change in physical activity, changes in mental health, vitamin D deficiency, increased air pollution, and increased incidence of infectious disease [26].

This study also identified variables associated with mortality in patients with cardiogenic shock requiring ECMO. In addition to previously described associated clinical variables, the study examined different socioeconomic and system level factors that might be affecting mortality in this population. Patients with specific chronic conditions such as diseases of the respiratory system or the genitourinary system were more likely to die. This was expected given that patients with multiple organ dysfunction syndrome require ventilator support or dialysis and are more likely to die. These findings are similar to previous studies where pre-ECMO predictive survival score (SAVE-score) in patients with refractory cardiogenic shock identified chronic renal failure as risk factor associated with mortality [24].

The study also examined the costs and impact associated with ECMO use. The mean total charges for cardiogenic shock visits with ECMO procedure was $589,610.5 (95%CI $512,270.0 – $666,950.9) per admission. This mean is more than 10 times the national mean cost of patients with cardiogenic shock post ST-Elevation Myocardial Infarction in 2010 ($45,625) [28]. The mean length of stay for the study population was 21.8 days (95%CI 19.0–24.7). Both the mean cost and mean length of stay are higher than what Maxwell et al. previously reported on resource use trends in ECMO between 1998 and 2009 where the mean total hospital charges was $344,009 ± $30,707 per admission and the mean length of stay was 18.3 ± 1.3 days [12]. Resources utilization with ECMO seem to be increasing without an associated increase in survival benefit. Future research is therefore needed to document trends in survival and the impact of ECMO use on outcomes of patients suffering from cardiogenic shock and from other conditions. ECMO is just one of several mechanical assist devices used in cardiogenic shock. Other devices include intra-aortic balloon pumps (IABP) and LV assist devices (LVADs). ECMO however, is the only intervention that compensates for both the right and left heart function as well as lung function. As such, ECMO decreases requirements for catecholamine, vasodilator, and mechanical ventilation in the treatment of cardiogenic shock and reduces the complications that are usually associated with these treatment modalities [18, 29]. ECMO use therefore holds promise for use in selected cardiogenic shock patients and further prospective studies are recommended.

The limitations of this study are directly related to structural features of the NEDS database. First, ICD-9 CM codes do not differentiate between VA or VV ECMO. This may be resolved with future use of ICD-10 CM codes. Second, information on the duration of ECMO use or timing of initiation is not available and costs are allocated to the whole hospital admission and not to a specific resource. Other limitations are related to the retrospective nature of the study. The quality and quantity of the data depends on the knowledge and experience of the coder, the completeness and accuracy of the patient record, and the state requirements. The study findings portray however trends in ECMO use for cardiogenic shock from a large sample from US based hospitals and can be generalized to other hospitals from similar settings.

## Conclusion

Mortality in adult patients with cardiogenic shock remains high despite ECMO use. Season of admission (other than Fall) and presence of specific comorbidities (Respiratory and genitourinary diseases) are associated with increased mortality in this population. Familiarity with patients’ characteristics and variables associated with mortality after ECMO use is important to improve outcomes further in cardiogenic shock. With rising costs and resources utilization, future research should focus on the impact of ECMO use on survival and on outcomes of patients with critical illnesses including but not limited to cardiogenic shock.

## Additional File

Additional File 1: A list of Equivalent ICD-9 CM codes and variable classification. (DOCX 106 kb)

## Abbreviations

AHRQ: Agency for Healthcare Research and Quality
CCS: Clinical Classifications Software
CI: confidence intervals
CPR: Cardiopulmonary Resuscitation
ECLS: extracorporeal life support
ECMO: Extracorporeal membrane oxygenation
ED: emergency department
ELSORIR: Extracorporeal Life Support Organization Registry International Report
HCUP: Healthcare Cost and Utilization Project
IABP: include intra-aortic balloon pumps
ICD: International Classification of Diseases
IRB: Institutional Review Board
LOS: length of stay
LVADs: LV assist devices
NEDS: Nationwide Emergency Department Sample
VA: veno-arterial
VV: veno-venous
